# Epigenetic Regulation of Kaposi’s Sarcoma-Associated Herpesvirus Latency

**DOI:** 10.3389/fmicb.2020.00850

**Published:** 2020-05-19

**Authors:** Mel Campbell, Wan-Shan Yang, Wayne W. Yeh, Chen-Hsuan Kao, Pei-Ching Chang

**Affiliations:** ^1^UC Davis Cancer Center, University of California, Davis, Davis, CA, United States; ^2^Institute of Microbiology and Immunology, National Yang-Ming University, Taipei, Taiwan

**Keywords:** Kaposi’s sarcoma-associated herpesvirus (KSHV), epigenetic, DNA methylation, histone modification, post-translational modification (PTM), long non-coding RNAs (lncRNAs)

## Abstract

Kaposi’s sarcoma-associated herpesvirus (KSHV) is an oncogenic *γ*-herpesvirus that infects humans and exhibits a biphasic life cycle consisting of latent and lytic phases. Following entry into host cells, the KSHV genome undergoes circularization and chromatinization into an extrachromosomal episome ultimately leading to the establishment of latency. The KSHV episome is organized into distinct chromatin domains marked by variations in repressive or activating epigenetic modifications, including DNA methylation, histone methylation, and histone acetylation. Thus, the development of KSHV latency is believed to be governed by epigenetic regulation. In the past decade, interrogation of the KSHV epitome by genome-wide approaches has revealed a complex epigenetic mark landscape across KSHV genome and has uncovered the important regulatory roles of epigenetic modifications in governing the development of KSHV latency. Here, we highlight many of the findings regarding the role of DNA methylation, histone modification, post-translational modification (PTM) of chromatin remodeling proteins, the contribution of long non-coding RNAs (lncRNAs) in regulating KSHV latency development, and the role of higher-order episomal chromatin architecture in the maintenance of latency and the latent-to-lytic switch.

## Epigenetic Regulation of Chromatin

“Epigenetics” refers to a heritable phenotype that changes the chromatin conformation and gene transcription without alteration of DNA sequence. Accurate epigenetic status is essential for normal development and maintenance of tissue-specific gene expression in mammals, and disruption of epigenetic regulation can cause aberrant gene expression and diseases, such as cancer. Different from genetic variation, epigenetics is a reversible mechanism that modifies the genome, and thus, repair of epigenetic lesions has been envisioned to be more feasible than correction of DNA mutations. Epigenetic therapies are therefore emerging as an active area of preclinical and clinical cancer research.

**FIGURE 1:**
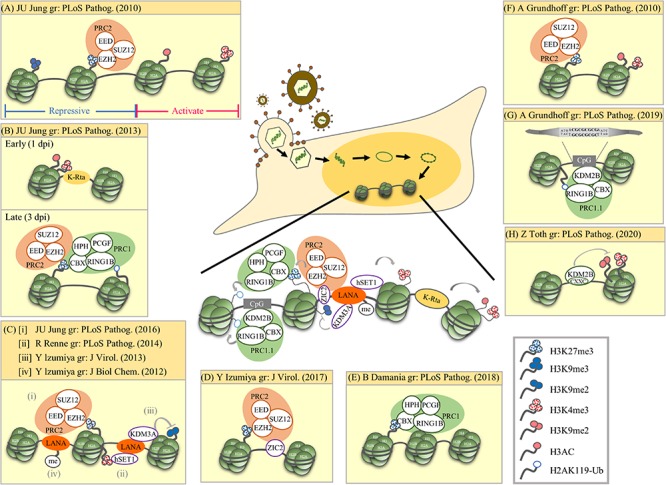
Summary of epigenetic modulation of the latent KSHV genome. The research group (gr) associated with each publication is listed. **(A)** Mutual exclusive localization of histone activation (H3K4me3 and H3-ac) and repressive (H3K27me3 and H3K9me3) marks. The PRC2 complex binds and catalyzes H3K27me3. **(B)** Biphasic euchromatin-to-heterochromatin transition following KSHV infection with initial K-Rta-mediated deposit of active histone marks (H3K4me3 and H3K27-ac). One to three days post-infection (dpi), active histone marks decline and a sequential deposit of H3K27me3 by PRC2, recruitment of PRC1, and deposit of H2AK119-Ub by PRC1. The sequential recruitment of modifying complexes converges to increase repressive histone marks (H3K27me3 and H2AK119-Ub). **(C)** LANA mediated recruitment of histone-modifying enzymes and deposition of corresponding histone marks. (i) LANA recruits PRC2 that potentially increase H3K27me3. (ii) LANA associates with H3K4 methyltransferase hSET1. (iii) LANA associates with H3K9me1/2 histone demethylase KDM3A. (iv) PRMT1-directed methylation of LANA increases its chromatin binding. **(D)** ZIC2 contributes to tethering of PRC2 on the KSHV genome, thus maintaining H3K27me3. **(E)** PRC1 involvement in maintaining nucleosomes on the latent KSHV genome. **(F)** Widespread presence of both active marks (H3K4me3 and H3K9/K14-ac) and repressive marks (H3K27me3) across the latent KSHV genome. **(G)** Direct binding of KDM2B to CpG islands recruits PRC1.1 on the latent KSHV genome. **(H)** KDM2B rapidly binds to the incoming viral DNA and limits the enrichment of activating histone marks on the RTA promoter favoring the downregulation of RTA expression. This early event occurs prior to the polycomb protein-regulated heterochromatin formation on the viral genome. **Summary:** Following *de novo* infection, KSHV K-Rta initiates the acquisition of the active histone marks H3K4me3 and H3K27-ac on the KSHV genome. After LANA is expressed, it mediates the increase of the repressive mark H3K27me3 and the active mark H3K4me3. H3K27me3 consequently recruits PRC1 and increases H2AK119-Ub on the KSHV genome. The cellular protein KDM2B may also help recruit PRCs to the KSHV genome.

**FIGURE 2:**
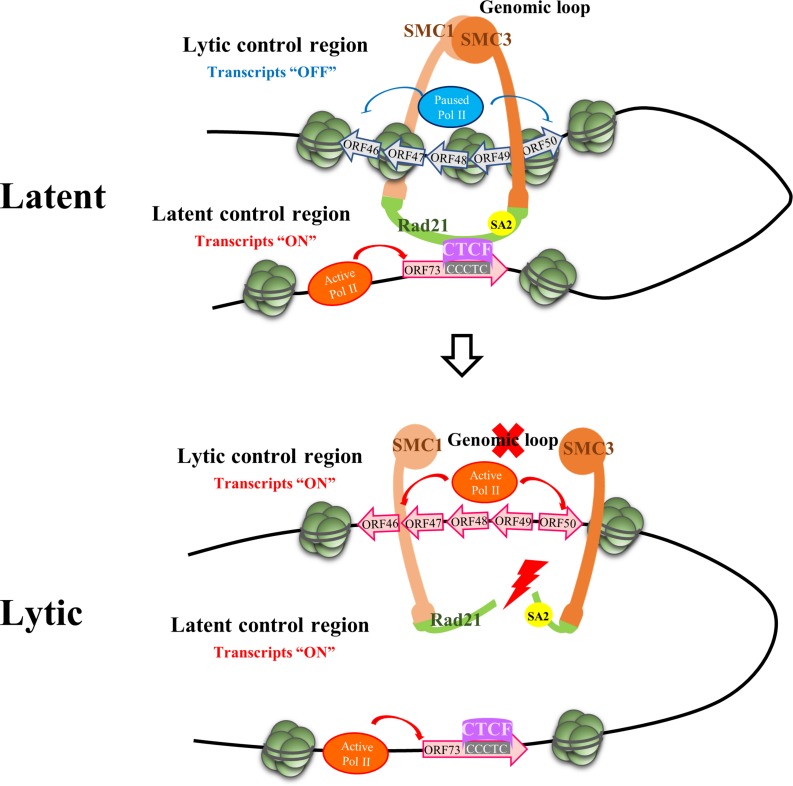
Episome Conformational Control of Latency. The figure depicts the region on the KSHV genome where chromatin contacts implicated in the maintenance of latency and the latent to lytic switch reside. Upper panel: The latency control locus contacts the lytic control region via a CTCF-dependent genomic loop during latency. CTCF sites in the latency control region are clustered within the first intron of ORF73. This looping mechanism permits latent gene expression while early lytic gene expression (i.e., K-Rta) is repressed. Lower panel: Opening of the cohesin ring during lytic reactivation results in loss of the genomic loop and facilitates RNA polymerase II (Pol II) activation at the early lytic locus resulting in K-Rta expression and induction of the lytic phase. The figure depicts RAD21 cohesin complex component (Rad21) cleavage as the initiating factor resulting in opening of the ring with subsequent loss of looping contacts. SMC, structural maintenance of chromosome proteins. CTCF, CCCTC-binding factor.

There are three primary interconnected epigenetic mechanisms (Figure 1), including (i) DNA methylation and hydroxymethylation, (ii) post-translational modifications (PTMs) of chromatin histone proteins, (iii) regulation by non-coding RNAs (ncRNAs), and a fourth relatively recently identified mode of epigenetic control, (iv) architectural/spatial epigenetics (Figure 2).

(i) Methylation of the 5th carbon on cytosine (5-mC) was the first identified, and one of the most well-studied epigenetic marks related to cancer (Bhattacharjee et al., 2016). DNA methylation is maintained by DNA methyltransferase 1 (DNMT1), an enzyme that prefers hemi-methylated DNA substrates, whereas DNMT3A and 3B are responsible for *de novo* methylation. 5-mC within CpG islands in mammalian promoter regions is associated with transcription repression and aberrant DNA methylation is a common lesion related to carcinogenesis (Baylin and Jones, 2011). Cytosine hydroxymethylation (5-hmC), a stable intermediate in 5-mC demethylation, was recently identified as a novel epigenetic modification on DNA in mammals (Richa and Sinha, 2014). 5-hmC seems to promote gene expression during active demethylation (Branco et al., 2011).
(ii) The N-terminal tails of histone proteins are post-translationally modified by acetylation, methylation, phosphorylation, ubiquitination, and SUMOylation, among other modifications (Kouzarides, 2007). The most studied histone modifications include acetylation and methylation. By *acetylating* the ε-amino group of lysine (Lys) using histone acetyltransferases (HATs), acetylation neutralizes the net positive charge on histones, leading to the unfolding of chromatin and exposure of negatively charged DNA to DNA-binding proteins, and consequently activation of gene transcription (Kouzarides, 2007). Histone deacetylases (HDACs) remove acetyl groups from histones and silence gene expression. Disrupting the balance between acetylation and deacetylation is linked to transcription dysregulation.

Histone methylation is more complex than acetylation in that both Lys and arginine (Arg) residues are known to be methylated. Arg can be mono- or di-methylated, with the latter in a symmetrical or asymmetrical manner, by protein arginine methyltransferases (PRMTs) (Pal and Sif, 2007). Lys has the potential to be mono-, di-, or tri-methylated by lysine methyltransferases (KMTs) (Klose and Zhang, 2007). In recent years, histone modification has gained attention due to the discovery of a large family of Jumonji C (JmjC) domain-containing histone lysine demethylases (KDMs) (Klose and Zhang, 2007). Histone methylation status is important in epigenetic regulation of gene expression and has been identified as a contributor to disease development.

(iii) Non-coding RNAs (ncRNAs) are RNA transcripts that do not encode proteins. Based on the length, ncRNAs are divided into two classes, (i) small ncRNAs (sncRNAs), with transcripts shorter than 200 nucleotides (nts), and (ii) long ncRNAs (lncRNAs), with transcripts longer than 200 nts that are devoid of protein-coding potential (Ponting et al., 2009; Fatica and Bozzoni, 2014), though some recent evidence shows that certain lncRNAs are able to encode small functional peptides (Nelson et al., 2016). In general, ncRNAs function to regulate gene expression at either the transcriptional or post-transcriptional level, and this regulation often involves components associated with epigenetic processes. Epigenetic-related ncRNAs include microRNAs (miRNAs) and lncRNAs. miRNAs are a group of sncRNAs of approximately 19–22 nts that inhibit target gene expression by binding to complementary regions of mRNAs and forming the miRNA-induced silencing complex (miRISC) (reviewed in Kim et al., 2009). The development of advanced next-generation sequencing (NGS) technology has revealed the presence of large amounts of lncRNAs in the human transcriptome. These RNAs share many common features with mRNAs, including (Bhattacharjee et al., 2016) 5′-methylguanosine cap, (Baylin and Jones, 2011) polyadenylation, (Richa and Sinha, 2014) RNA polymerase II transcription, and (Branco et al., 2011) splicing (Derrien et al., 2012). However, lncRNAs are generally expressed at a lower level and displayed higher tissue specificity than mRNAs (Fatica and Bozzoni, 2014). Emerging evidence suggests that lncRNAs are involved in regulating multiple biological processes through mechanisms including transcriptional (Dimitrova et al., 2014), post-transcriptional (Yoon et al., 2013; Hu X. et al., 2014), and epigenetic (Khalil et al., 2009; Gupta et al., 2010; Tsai et al., 2010) regulation.
(iv) Architectural/spatial epigenetics considers the three-dimensional (3D) structure of a genome and its impact on gene expression and other nuclear activities. How the 3D organization of a genome operates with the addition of dynamics across time and its relationship to nuclear processes including transcription, DNA replication, and chromosome segregation are included in this class of mechanisms (Dekker and Mirny, 2016; Dekker et al., 2017).

## The KSHV Genome and Latency

The human *γ*-herpesvirus Kaposi’s sarcoma-associated herpesvirus (KSHV, also known as human herpesvirus type 8, HHV-8) is one of the seven recognized human oncogenic viruses and has been linked to Kaposi’s sarcoma (KS) (Scadden, 2003), primary effusion lymphoma (PEL) and Multicentric Castleman’s disease (MCD) (Wen and Damania, 2010), and an inflammatory syndrome known as KSHV inflammatory cytokine syndrome (KICS) (Uldrick et al., 2010). KSHV is a linear double-stranded DNA virus with genome size of approximately 165–170 kb consisting of a unique coding region (∼145 kb) that encodes ∼90 viral proteins and many non-coding RNAs and is flanked by long terminal repeats (TRs) (Renne et al., 1996; Russo et al., 1996). After entry into host cells, viral genomes are circularized by joining of GC-rich TRs. The viral genomes associate with cellular histones and form an extrachromosomal circular viral episomes. Establishment of latency in infected cells is a common tactic that herpesviruses employ to prevent elimination by the host immune response and to establish lifelong persistent infections. In order to establish and maintain latency, KSHV has acquired different strategies to hijack the host epigenetic machineries to help viral episomes form a heterochromatin structure that restricts viral gene expression to a few genes during latency.

## DNA Methylation Vs. KSHV Latency

DNA methylation on CpG islands is associated with gene silencing. It has been shown that the DNA methyltransferase inhibitor 5-Azacytidine (5-AzaC) is a stimulator of KSHV lytic reactivation (Chen et al., 2001), suggesting the involvement of DNA methylation in maintaining KSHV latency. In 2010, Günther and Grundhoff used MeDIP-tiling microarrays to detect global DNA methylation patterns on the KSHV genome following *de novo* infection (Gunther and Grundhoff, 2010). They showed extensive DNA methylation on KSHV latent genomes with the exception of the latency-associated locus. Surprisingly, global viral DNA methylation patterns were shown to develop slower than latency-specific histone modifications (Gunther and Grundhoff, 2010), indicating that DNA methylation is involved in the establishment or maintenance of latency at a later stage relative to histone modifications. Therefore, DNA methylation was proposed to reinforce the inhibition of viral gene expression conferred by repressive histone modifications during *de novo* infection. This notion was supported by a report from Darst et al. (2013). Using methylation accessibility probing for individual templates (MAPit) to map CG methylation on chromatin structure in latent KSHV episomes, these authors suggested that DNA methylation can restrict viral reactivation by chromatin compaction (Darst et al., 2013).

It is important to note that both Gunther and Grundhoff (2010) and Darst et al. (2013) observed that latency could be established independent of DNA methylation at the KSHV replication and transcription activator (K-Rta, ORF50) locus. Although the exact role of DNA methylation and viral latency is unresolved, gradual methylation of the KSHV genome may be important for long-term latency within the host but could also reflect the consequences of a host defense mechanism. These results support an idea originally proposed by Grundhoff and Ganem (2004) for the necessity of infrequent episodes of lytic replication for long-term KSHV persistence in the host.

## Histone Modifications Vs. KSHV Latency

In 2010, two ChIP-on-Chip studies initiated the analysis of global chromatin marks across KSHV latent genomes (Gunther and Grundhoff, 2010; Toth et al., 2010). In one report, Toth et al. revealed a mutually exclusive pattern of active and repressive histone marks on the KSHV genome, in which the active marks H3K4me3 and H3-ac are present in some parts of the KSHV genome whereas repressive marks H3K27me3 and H3K9me3 are located in other parts. The same report also showed the colocalization of EZH2, the H3K27me3 methyltransferase of Polycomb repressive complex 2 (PRC2), with H3K27me3 on KSHV latent genomes, suggesting a role of PRC2 in mediating deposition of H3K27me3 on the viral genome during latency (Figure 1A; Toth et al., 2010). Following these observations, Toth et al. (2013) found a biphasic euchromatin-to-heterochromatin transition on the KSHV genome upon *de novo* infection. Initially (<1 day post-infection, dpi) with the help of K-Rta, KSHV genomes rapidly acquired the active histone marks H3K4me3 and H3K27-ac. This was followed by (1st to 3rd dpi) sequential deposition of repressive histone marks H3K27me3 by PRC2 and H2AK119-Ub by Polycomb repressive complex 1 (PRC1). It is believed that binding of CBX in PRC1 to H3K27me3 deposited by PRC2 helps recruit PRC1 to the KSHV genome and RING1B in PRC1 ubiquitinated H2A at K119 (Figure 1B). In 2016, Toth et al. (2016) further revealed the potential of KSHV latency-associated nuclear antigen (LANA), a KSHV latent protein that is expressed very early after *de novo* infection, in mediating the recruitment of PRC2 onto the KSHV genomes during *de novo* infection (Figure 1Ci). Moreover, Hu et al. showed that KSHV LANA interacts and recruits the H3K4me3 methyltransferase hSET1 onto the KSHV genome (Figure 1Cii). This result explains the potential underlying mechanism for the deposition of H3K4me3 on latent KSHV genomes (Hu J. et al., 2014). Kim et al. (2013) showed that KSHV LANA also interacts and recruits the H3K9me1/2 demethylase KDM3A onto the KSHV genome (Figure 1Ciii). This suggests a mechanism for maintaining low H3K9 methylation on latent KSHV genomes. In addition to LANA, Lyu et al. (2017) also identified ZIC2 as a novel cellular protein that contributes to tethering PRC2 on the KSHV genome, thus maintaining H3K27me3 (Figure 1D). In 2018, Hopcraft et al. (2018) showed that ubiquitination of H2A at K119 by PRC1 is essential for prevention of nucleosome depletion on the KSHV genome and for maintaining KSHV latency (Figure 1E).

Gunther and Grundhoff (2010) also showed the presence of both the active marks H3K4me3 and H3K9/K14-ac and the repressive mark H3K27me3 on the KSHV genomes following *de novo* infection (Figure 1F). Following these observations, Gunther et al. (2014) also found that LANA may increase H3K27me3 deposition by inhibiting soluble Sp100, a negative regulator of PRC2 recruitment, through SUMOylation of Sp100. Recently, this group performed a comprehensive epigenome analysis of the KSHV genome and found the CpG motif as a cis-acting sequence for KDM2B, which consequently recruits Polycomb repressive complexes (PRCs) (Figure 1G; Gunther et al., 2019). Interestingly, a recent report from Naik et al. (2020) describes siRNA screening and time course ChIP experiments showing that early deposition of KDM2B on the KSHV genome limits the enrichment of the active marks H3K4me3 and H3K36me2 and helps in the maintenance of viral latency (Figure 1H). The binding of KDM2B prior to PRC-regulated heterochromatin supports the potential role of KDM2B in the recruitment of PRCs to viral DNA following *de novo* initial infection.

In summary (Figure 1, center), genome-wide studies of histone modifications on the KSHV latent genome revealed a mutually exclusive pattern of active and repressive histone marks, in which active marks, such as H3K4me3 and H3Ac, are located in certain parts of the KSHV genome, whereas repressive marks, such as H3K27me3 and H3K9me3, are located in other parts (Toth et al., 2010; Gunther et al., 2014, 2019). However, a bivalent chromatin structure that consists of both an active mark H3K4me3 and a repressive mark H3K27me3 was also identified in several promoter regions encoding immediate early (IE) (such as K-Rta) and early (E) genes (Gunther and Grundhoff, 2010; Toth et al., 2010; Jha et al., 2014; Lyu et al., 2017). Mechanistically, KSHV K-Rta may help facilitate the initial acquisition of the active histone marks H3K4me3 and H3K27-ac on the KSHV genome (Toth et al., 2013). Since KSHV LANA participates in the maintenance of latency through targeting KSHV K-Rta (Lu et al., 2006), after LANA is expressed, methylation of LANA by protein arginine methyltransferase 1 (PRMT1) may help stabilize LANA on the KSHV genomes (Figure 1Civ; Campbell et al., 2012), which consequently recruit PRC2 (Toth et al., 2016) and hSET1 (Hu J. et al., 2014) onto the KSHV genomes and mediate the increase of the repressive mark H3K27me3 and the active mark H3K4me3, respectively. H3K27me3 deposited by PRC2 may consequently aid the recruitment of PRC1, increasing H2AK119-Ub on the KSHV genome (Toth et al., 2013) and maintain nucleosomes on viral chromatin (Hopcraft et al., 2018). In addition to viral proteins, cellular factors may also be involved in recruitment. The direct binding of the PRC component KDM2B to CpG islands may also help in the recruitment of PRCs to the KSHV genome (Gunther et al., 2019; Naik et al., 2020).

## PTMs Vs. KSHV Latency

PTMs, including phosphorylation, ubiquitination, and Small Ubiquitin-related MOdifier (SUMO) modification, were initially identified as reversible protein modifications that regulate signal transduction. Among the PTMs, accumulating evidence suggests that the SUMO system plays an important role in regulating chromatin organization and transcription (Cubenas-Potts and Matunis, 2013). In addition, SUMOylation is also required for the assembly and disassembly of promyelocytic leukemia protein (PML) bodies, a host antiviral system that was found to mediate herpesvirus latency (Sewatanon et al., 2013). It is not surprising that KSHV has exploited the SUMOylation system to modulate its latency. Among viral proteins being SUMOylated, the KSHV IE proteins K-bZIP is a SUMO E3 ligase (Chang et al., 2010) and K-Rta is a SUMO-targeting ubiquitin ligase (STUbL) (Izumiya et al., 2013).

As SUMO modifications regulate chromatin organization, we showed that SUMOylation of the chromatin binding protein Krüppel-associated box domain-associated protein-1 (KAP-1) is essential for its association with the KSHV genome and for maintaining viral latency. Phosphorylation of KAP-1 by KSHV vPK (ORF36) counteracts KAP-1 SUMOylation-dependent binding and thereby facilitates viral reactivation (Chang et al., 2009). Binding of SUMOylated KAP-1 with LANA through a SUMO-SIM (SUMO interacting motif) interaction maintains KSHV latency (Cai et al., 2013). In addition, inhibition of SUMO/sentrin-specific peptidase 6 (SENP6) expression by LANA was also found to be important in the establishment of latency (Lin et al., 2017). These results together support the potential role of SUMO in maintaining latent KSHV genomes.

In 2015, the genome-wide landscape of SUMO paralog modifications on the KSHV genome was revealed using ChIP-seq assays. The results showed similar SUMO-1 and SUMO-2/3 binding patterns on KSHV latent viral genomes and detailed a significant increase of SUMO-2/3 deposition upon reactivation (Yang et al., 2015). Mechanistically, the KSHV SUMO E3 ligase K-bZIP interacted with (Chang et al., 2011) and SUMOylated (Yang et al., 2017) JMJD2A, a Jumonji domain containing H3K9me3 demethylase. This SUMO modification stabilized JMJD2A on chromatin and therefore maintained the KSHV genome with low levels of H3K9me3 (Gunther and Grundhoff, 2010; Jha et al., 2014; Gunther et al., 2019). This action prevents the formation of heterochromatin on KSHV episomes and maintains the viral genome in a poised state that is prepared for rapid activation (reviewed in Chang and Kung, 2014). These results were supported by a previous report from Hilton et al. (2013) that found open chromatin in both transcriptionally active and inactive loci in latent KSHV episomes. Altogether, these results suggest that PTM of chromatin remodeling proteins is another mechanism that contributes to the epigenetic regulation of KSHV latency.

## Non-Coding RNAS Vs. Kshv Latency

Gene expression profiling of the KSHV life cycle using real-time PCR, oligonucleotide arrays, and Northern blotting (Dittmer, 2003; Chandriani and Ganem, 2010) has detected pervasive transcription throughout the KSHV viral genome, indicative of a complex viral transcriptome. These studies were later confirmed and expanded by comprehensive functional genomic approaches (Arias et al., 2014; Bruce et al., 2017). KSHV transcriptional complexity includes the expression of non-coding RNAs including miRNAs (Cai et al., 2005; Samols et al., 2005; Grundhoff et al., 2006) and lncRNAs (Schifano et al., 2017). A total of 12 KSHV pre-miRNAs that can evolve into 25 mature miRNAs were identified by four groups in the years 2005–2006 [review in (Qin et al., 2017)]. KSHV miRNAs are clustered in the latency-associated locus of the KSHV genome Gottwein et al. (2006). By using a KSHV miRNA deletion mutant, Lu et al. detected several epigenetic changes in the KSHV genome upon loss of KSHV miRNAs. These included a decrease in DNA methylation, a decrease in the repressive mark H3K9me3, and an increase in the active mark H3-ac throughout KSHV genome (Lu et al., 2010). They concluded that KSHV miRNAs are involved in maintaining the latent genome of KSHV by targeting multiple pathways, including an indirect effect on K-Rta and a direct effect on a cellular target Rb-like protein 2 (Rbl2). Rbl2 is known as a regulator of epigenetic reprogramming and loss of Rbl2 resulted in derepression of DNMT3A and 3B that consequently led to an increase in KSHV and host DNA methylation (Lu et al., 2010). Other KSHV miRNAs targeting K-Rta with modest effects on the latent-to-lytic switch have also been reported (Bellare and Ganem, 2009; Ziegelbauer et al., 2009).

A succession of studies using a variety of experimental approaches have described the existence of at least 16 potential KSHV lncRNAs, and these have been nicely summarized by Schifano et al. (2017). KSHV lncRNAs have been characterized to varying levels of detail with the best studied species as the 1.1 kb polyadenylated nuclear RNA (PAN RNA), which was first described in 1996 (Sun et al., 1996; Zhong et al., 1996). The PAN RNA promoter is a direct target of K-Rta (Song et al., 2001, 2002; Bu et al., 2008). Thus, its expression is highly increased (>1000-fold) upon reactivation (accounting for 65% and > 80% of the KSHV reads at 8 h and 24–72 h, respectively, post-induction of reactivation) with early kinetics (Rossetto et al., 2013; Arias et al., 2014; Bruce et al., 2017). PNA RNA persists into the late stage of lytic replication and is packaged into virions (Bechtel et al., 2005). In PEL cell lines, PAN levels are capable of reaching an estimated 1–5 × 10^5^ copies per cell (Sun et al., 1996; Song et al., 2001) and accumulate as one of the most abundant viral RNA species present in the infected cell during lytic reactivation.

As a lytic transcript, how could PAN RNA affect viral latency? By using chromatin isolation by RNA purification (ChIRP) assay, Rossetto et al. (2013) demonstrated the occupancy of PAN RNA at multiple sites on the KSHV genome, including the K-Rta promoter region. PAN RNA binding was proposed to recruit the cellular factors JMJD3 and UTX, which are H3K27me3 demethylases and the H3K4me3 methyltransferase MLL2. This consequently decreased the repressive mark H3K27me3 and increased the activation mark H3K4me3 on the K-Rta promoter followed by disruption of viral latency (Rossetto and Pari, 2012). The interaction of PAN RNA with KSHV DNA polymerase processivity factor (ORF59) (Rossetto and Pari, 2011) may also contribute to the function of PAN RNA in activation of gene expression during the lytic phase. However, contrasting results were also found by Rossetto et al. (2013) using ChIRP. They demonstrated occupancy of PAN RNA on the KSHV genome and its association with PRC2 components EZH2 and SUZ12, which in turn increased the repressive mark H3K27me3 that acts to repress gene expression. These data together suggest that PAN RNA may function in either positive or negative epigenetic regulation depending on cell context (reviewed in Campbell et al., 2014b; Rossetto and Pari, 2014).

Interaction of PAN RNA with additional viral factors has also been examined. Through direct interaction with LANA, PAN RNA dissociates LANA from the KSHV genome and disrupts viral latency (Campbell et al., 2014a). Using an alternative to ChIRP known as capture hybridization analysis of RNA targets (CHART) and nuclear fractionation studies, Withers et al. showed that KSHV PAN RNA, although nuclear, was not associated with chromatin (Withers et al., 2018). These results suggested chromatin-independent activities of PAN RNA. Thus, in contrast to results obtained by others (Rossetto and Pari, 2012, 2014; Rossetto et al., 2013; Campbell et al., 2014a), modulation of gene expression at specific viral or host chromatin loci was not considered the pertinent function of PAN RNA. Rather, PAN RNA was proposed to function in nuclear mRNA export of late viral mRNAs. Taken together, current research has suggested several functions for PAN RNA, which can be generally classified as chromatin-associated or chromatin-independent activities, although the exact role of this lncRNA in the viral life cycle is still unclear.

## Role of Higher-Order Episomal Organization in Latency

The development of high-resolution chromosome conformation capture (3C)-based methodologies (Dekker and Mirny, 2016) has established that eukaryotic genomes are arranged or folded in a specific manner within the three-dimensional nuclear space. Crucial to this arrangement were the discoveries concerning the roles of CCCTC-binding factor (CTCF) and cohesins (containing structural maintenance of chromosomes; SMC protein subunits) in the dynamics of chromatin organization (Merkenschlager and Nora, 2016; Braccioli and de Wit, 2019). Since circularized KSHV episomes resemble host chromatin in terms of decoration with host histones which are substrates for host chromatin modifiers, it was not too surprising that cellular epigenetic machinery has been implicated in control viral chromatin architecture and viral latency. The genome-wide localization of CTCF and cohesins along the KSHV genome, including the identification of a series of highly enriched sites in the latency control region, was first reported by Stedman et al. (2008). Subsequent studies confirmed the existence of approximately 25 major CTCF binding sites on the KSHV genome, most of which exhibited colocalized cohesin binding although the overall amount of cohesin binding was less (Li et al., 2014). Kang et al. (2011) reported on the existence of a CTCF/cohesin-mediated genomic looping that coordinates KSHV latent and lytic gene expression. 3C and other chromosome conformation assays were used to probe latent KSHV cross-linked chromatin for contacts involving the latency control region and other positions on the KSHV genome. Multiple loops between the latency control region and other KSHV genomic locations were detected, including a high frequency of contacts between a CTCF/cohesin binding site in the latency control region with (i) the 3′ end of the LANA coding region and (ii) the 5′ promoter region of ORF50 (Kang et al., 2011). This pair of loops were suggested to insulate latent and lytic viral gene and coordinate their expression. This arrangement ensures the repression of lytic transcription during latency, while latency transcription occurs efficiently. Moreover, these loops were reduced or eliminated during lytic reactivation, indicating that these contacts are dynamic. Epigenetic changes including RNA Pol II enrichment throughout the early gene locus spanning ORF45–50 transcripts were also noted during cohesin depletion experiments (Chen et al., 2012; De Leo et al., 2017). Additional studies that focused on DNA looping between the latent and lytic control regions found that disruption of this loop also accompanies lytic reactivation induced by ER stress-mediated Rad21 cleavage (De Leo et al., 2017) or lytic induction via Bromodomain and Extended Terminal (BET) protein inhibitor treatment (Chen et al., 2017). Together, these studies have created a model whereby the CTCF/cohesin-dependent genomic linkage between the latency and lytic control regions is necessary for preservation of the latent state. Any structural perturbation to this latent genomic conformation facilitates the switch to the lytic state through locus-specific association and activation of RNA Pol II (Figure 2). Although this model highlights effects on gene expression via the topological organization of viral chromatin, local and direct effects of CTCF and cohesion on promoter activity are also likely (Dorsett and Merkenschlager, 2013; Merkenschlager and Odom, 2013). Cohesin may regulate RNA Pol II pausing (Fay et al., 2011) and CTCF binds RNA Pol II may facilitate RNA Pol II recruitment and elongation (Chernukhin et al., 2007; Pena-Hernandez et al., 2015). Together, this complex behavior emphasizes the multifactorial nature of the mechanisms CTCF and cohesin may utilize to regulate KSHV gene expression.

The looping/conformation model described above has only been studied in the context of latently infected cells and, as such, attempts to explain the maintenance of latency and the latent-to-lytic switch. However, it is currently not known how initial episomal chromatin conformations are established following *de novo* infection. Although Toth et al. (2017) have reported that CTCF and cohesin rapidly associate with incoming KSHV genomes following *de novo* infection, detailed mechanisms of the initial high-order structuring and nuclear residences of KSHV episomes are unknown.

## Conclusion and Future Prospects

Genome-wide analysis of KSHV episomes have revealed distinct temporal and spatial chromatin modification patterns on the viral genome. In summary, following *de novo* infection, the KSHV IE protein K-Rta may help viral episomes acquire active histone marks (Toth et al., 2013) and cellular factors such as KDM2B may function to limit the enrichment of active histone marks (Naik et al., 2020). The binding of KDM2B to viral episomes may consequently help recruit the PRCs and the deposition of repressive marks. These modifications prevent the virus lytic cycle and promote the establishment of viral latency. Following these events, the latent viral protein LANA is expressed, which helps recruit PRC2 (Toth et al., 2016) and hSET1 (Hu J. et al., 2014) onto the KSHV genomes and mediates the formation of site-specific bivalent chromatin structures. Expression of KSHV miRNAs during latency may also assist with the deposition of DNA methylation and the heterochromatin mark H3K9me3 (Lu et al., 2010). However, the SUMOylation of H3K9me3 demethylase JMJD2A by the KSHV lytic protein K-bZIP maintains JMJD2A on the KSHV genome and prevents the formation of heterochromatin (Yang et al., 2015, 2017), resulting in maintenance of the latent KSHV episome in a poised state that is ready for rapid reactivation. The expression of the KSHV lncRNA PAN during the early stages of lytic reactivation helps recruit multiple histone modification enzymes to the viral genome and further disrupts viral latency (Rossetto and Pari, 2012). As the switch from latency to lytic replication is essential for viral survival and spread, the role of KSHV lncRNAs in maintaining a balanced viral chromatin state to persist within the host is clearly an important topic for future research. In addition, identifying cellular lncRNAs that are up- or down-regulated during both the lytic and latent states of KSHV infection is also an interesting question to explore in terms of establishing how cellular lncRNAs influence the KSHV life cycle. Spatial and architectural conformation of KSHV episomal chromatin is a relatively understudied feature of KSHV biology; however, episome conformational contacts have been observed to be a dynamic feature of KSHV chromatin at both the focused level of 3C (Kang et al., 2011; Chen et al., 2017; De Leo et al., 2017) and at the global level of Capture Hi-C (Campbell et al., 2018) suggestive of potential roles in the viral life cycle.

The compartmentalization of the epigenetic marks on the KSHV genome might be beneficial for transcription regulation of viral gene expression during its distinct life cycle. The well-organized DNA methylation and different histone modification patterns on the KSHV genome reflects the precise recruitment of cellular chromatin modifying complexes employed by the virus. KSHV latency is essential for persistent infection as well as the development of KSHV-associated malignancies. In recent years, more and more epigenetic modifications and the corresponding modifying enzymes have been identified. Drugs targeting epigenetic modification enzymes have now evolved into a potential viable strategy for controlling persistent viral infections (Nehme et al., 2019). Thus, elucidating the epigenetic regulators involved in establishing KSHV latency may be a new avenue for pharmacological control of KSHV-associated diseases.

## Author Contributions

MC wrote and edited the manuscript. W-SY helped writing the manuscript. C-HK constructed the figures. P-CC worked on the constructs and the manuscript writing. WY contributed to the final revision with modification of manuscript and Figure 1 and compose Figure 2.

## Conflict of Interest

The authors declare that the research was conducted in the absence of any commercial or financial relationships that could be construed as a potential conflict of interest.
